# A Programmable Finite-Replicated Organism Framework for Balanced Safety and Functionality

**DOI:** 10.3390/life15091381

**Published:** 2025-09-01

**Authors:** Mengyuan Wang, Pei Du, Fankang Meng, Wenhui Zhang, Yanhui Xiang, Qiong Wu, Chunbo Lou

**Affiliations:** 1 State Key Laboratory of Green Biomanufacturing, MOE Key Lab. Bioinformatics, Center for Synthetic and Systems Biology, School of Life Sciences, Tsinghua University, Beijing 100084, China; wangmy5600@163.com; 2 CAS Key Laboratory of Pathogen Microbiology and Immunology, Institute of Microbiology, Chinese Academy of Sciences, Beijing 100101, China; dupeicn@gmail.com (P.D.); mengfankang1994@gmail.com (F.M.); 3 Center for Cell and Gene Circuit Design, State Key Laboratory of Quantitative Synthetic Biology, Shenzhen Institute of Synthetic Biology, Shenzhen Institutes of Advanced Technology, Chinese Academy of Sciences, Shenzhen 518055, China; zhangwenhui1994@126.com

**Keywords:** synthetic biology, live-attenuated vaccine, finite-replicated organisms, noncanonical amino acid, essential gene, toxin–antitoxin system

## Abstract

Live-attenuated vaccines face a critical challenge in balancing immunogenicity with safety. To address this, we engineered programmable finite-replicated organisms (FROs) by depositing a limited number of indispensable components (such as noncanonical amino acids, ncAAs) within the cell, consuming the coenabling precise control of bacterial replication capability while preserving antigenic breadth. Two strategies were adopted to achieve the following purposes: (1) encoding ncAA in essential genes; (2) encoding ncAA in antitoxin of toxin–antitoxin (TA) systems. As noncanonical amino acids, 3,5-dichlorotyrosine (Cl2Y) was encoded by the amber codon (TAG) and inserted into the essential genes (e.g., *serS*, *murG*, and *dnaA*) or antitoxin genes. After optimizing expression and the number of amber codons in the storage genes, the FRO cells can grow up to six generations, achieving amplification approaching 100 times after depletion of the ncAA in the growth medium. The escape frequencies are 10^−5^ to 10^−7^, which need to be optimized by combining multiple storage genes in the same genome in the future. This work holds the potential to amplify the amounts of antigens for vaccines, potentially accelerating the development of next-generation vaccines against antibiotic-resistant threats.

## 1. Introduction

The emergence and re-emergence of infectious diseases pose significant challenges to public health and economic stability [1,2]. Vaccination stands out as the most effective solution for preventing the spread of infectious diseases [2,3]. However, the current types of vaccines, including live-attenuated, inactivated, protein subunit vaccines, and mRNA vaccines, each have their advantages and face distinct challenges [3,4]. Specifically, whole-pathogen vaccines such as live-attenuated vaccines provide a comprehensive array of antigens that closely mimic natural infections [5], effectively eliciting a broad range of immune responses across both humoral and cellular immunity, but the replicative nature of these vaccines often brings concerns in safety. The *Brucella abortus* 104M strain exemplifies this dilemma: despite clinical deployment since 1965 as a human vaccine, its residual pathogenicity restricts application to high-risk scenarios requiring stringent risk–benefit analysis [6,7]. Alternative platforms mitigate safety risks yet frequently encounter suboptimal immunogenicity or antigenic targeting limitations. This enduring challenge highlights the central paradigm in modern vaccinology: optimizing the delicate equilibrium between protective immunity and biological containment.

Faced with this dilemma, it would be ideal if one vaccine could provide optimal immunogenicity by presenting a full set of antigens and maintaining the replication capacity, while ensuring safety by precisely controlling its replication. Such a balance would maximize the immune response while minimizing risks, especially in vulnerable populations. To achieve this, a new class of organisms exhibiting a strictly regulated replication capacity needs to be developed, namely Finite-Replicated Organisms (FROs). An FRO-based vaccine would need three key components: (i) a robust “kill switch” that can terminate the organism’s replication in response to a specific signal, ensuring safety; (ii) a sensitive “tuner” that regulates the number of generations the organism can replicate by sending the “kill signal,” allowing for control over the vaccine’s immunogenicity; and (iii) a “kill signal” coordinating the “tuner” and “kill switch” that is orthogonal to all internal signals under physiological conditions to provide a fail-safe mechanism for vaccine safety. These components, constructed with existing systems or novel synthetic biology tools, could build an innovative FRO framework that offers escape-proof and programmable regulation of growth, laying the foundation for FRO-based vaccines that combine the effectiveness of whole-pathogen vaccines and the safety of subunit vaccines.

Previous research has provided building blocks for the design of FROs. For example, the mutated essential genes and an orthogonal translation system composed of an aminoacyl-tRNA synthetase (aaRS) and tRNA pair in the genetically recoded organisms (GROs) offer a compelling framework, as such a system makes the GROs auxotrophic and dependent on the presence of noncanonical amino acids (ncAAs) to survive [8,9,10]. These ncAA-dependent mutations in essential genes can function effectively as a “kill switch” for FROs. Importantly, as ncAAs are not naturally found in environments or living organisms [11], the lack of ncAAs in a host would ensure that the FRO stops replicating once the supply of ncAAs is exhausted, which makes ncAA an ideal “kill signal” that offers robust control over the organism’s survival and replication. In parallel, toxin–antitoxin (TA) systems present an alternative strategy for constructing FROs. TA systems typically consist of a toxin that inhibits growth and an antitoxin that neutralizes the toxin’s effects [12]. In this context, the toxin could act as the “kill switch”, while the antitoxin could function as the “tuner” by controlling the neutralization of the toxin. These systems allow fine-tuning of replication, creating an FRO that can be engineered to stop replication after a set number of generations, depending on the antitoxin’s action. Moreover, TA systems are abundant within the genomes of most bacterial species [12,13]. The diversity of TA systems provides a large reservoir for excavating synthetic parts, making TA systems an excellent resource for further designing FRO-based vaccines.

In this study, we aimed to engineer an ncAA-dependent FRO and demonstrated its ability for programmable finite replication. Using *E. coli* as a model organism, we constructed two sets of suicide and rescue modules that functioned as the “kill switch” and “tuner”, respectively. These modules were developed using two distinct strategies: essential gene manipulation and a toxin–antitoxin system. Both strategies successfully achieved finite replication of the engineered organisms. Moreover, we demonstrated that the number of generations could be programmed by adjusting ribosome binding site (RBS) strength or by varying the number of inserted ncAAs. Notably, both sets of suicide and rescue modules were dependent on the same ncAA, 3,5-dichlorotyrosine (Cl2Y), as the “kill signal”, which could be combined within a single FRO to further reduce the escape frequency and improve programmability. We believe this FRO represents an innovative framework for designing vaccines with balanced safety and effectiveness.

## 2. Materials and Methods

### 2.1. Strains and Culture Conditions

The *E. coli* C321.ΔA exp strain (Addgene #87359) (Lajoie MJ, 2013) was cultured in Luria–Bertani medium (10 g/L NaCl, 10 g/L tryptone, 5 g/L yeast extract) supplemented with antibiotics: ampicillin (100 μg/mL), kanamycin (50 μg/mL), chloramphenicol (25 μg/mL), and spectinomycin (50 μg/mL). Solid media contained 2% agar. Gene expression was induced with 1 mM IPTG (pTac promoter), 100 ng/mL anhydrotetracycline (pTet), or 0.2% L-arabinose (pBAD), as necessary.

### 2.2. Plasmid Construction

Plasmids used in this study are listed in Table S1. Plasmids carrying orthogonal translation systems for 3,5-dichlorotyrosine (Cl2Y) and p-azido-L-phenylalanine (pAzF) were constructed as previously described [8,14]. The DNA fragments of aminoacyl-tRNA synthetase (aaRS) and tRNA pair used for plasmid construction were generated by PCR amplification with PrimeSTAR Max DNA Polymerase (Takara) and assembled by the Gibson Assembly cloning kit (New England Biolabs, Ipswichu, MA, USA) into a specific vector with p15A ori. Plasmids were extracted using the TIANGEN plasmid extraction kit.

The rescue module (pTac promoter, ampicillin resistance) contained either RFP with eight amber codons or antitoxin genes with TAG insertions. Suicide modules (pBAD promoter, chloramphenicol resistance) co-expressed toxins and orthogonal translation system (OTS) components. RBS sequences were designed using the RBS Calculator [15] (Table S2). Other standard molecular cloning procedures, including plasmid isolation (TIANGEN, Beijing, China), DNA purification (Biomed, Shenzhen, China), and restriction enzymes and ligation kits (Thermo-Fisher, Beijing, China), were obtained commercially.

All plasmids generated in this study were constructed through conventional molecular cloning approaches. DNA inserts were PCR-amplified from plasmid templates using PrimeSTAR Max DNA Polymerase (Takara), while core vector backbones were engineered via Gibson Assembly Master Mix (NEB). Custom DNA sequences and oligonucleotide primers were commercially synthesized by Dynegene Biotech (Shanghai, China) and Ruibiotech (Beijing, China), respectively.

### 2.3. Quantitative Detection of Aminoacyl-tRNA Synthetase Efficiency

The efficiency of noncanonical amino acid (ncAA) incorporation was assessed via fluorescence level cultured under ncAA-supplemented conditions. The pAzF and Cl2Y orthogonal translation systems (OTSs) were transcriptionally controlled by the arabinose-inducible pBAD promoter and the ncAA/tRNA_CUA_ was driven by proK promoter. These recombinant strains harbored an episomal GFP reporter construct containing two strategically positioned in-frame TAG codons downstream of the start codon. Successful suppression of these amber codons by the OTSs through ncAA incorporation (specifically pAzF or Cl2Y) generated full-length fluorescent proteins, thereby validating both the incorporation fidelity of the ncAAs and the functional specificity of the translation systems.

### 2.4. Flow-Cytometry (FCM) Measurement

For FCM-based fluorescence quantification, *E. coli* cells were cultured in 96-deep-well plates at 37 °C in LB medium. Inducers (IPTG, arabinose, or aTc) were supplemented at specified concentrations during logarithmic growth. Following 24 h of incubation, cells from individual wells were resuspended in phosphate-buffered saline (PBS) at a 1:20 dilution ratio. Fluorescent reporters (GFP and RFP) were quantified using an LSRFortessa flow cytometer (BD Biosciences, Franklin Lakes, NJ, USA) equipped with a 96-well HTS injector. For GFP detection, cells were excited with a 488 nm laser, and fluorescence was collected through a 530/30 nm bandpass filter (FITC channel). RFP signals were acquired using 561 nm laser excitation with emission captured via a 610/20 nm bandpass filter (PE channel).

### 2.5. Strain Construction of Essential Gene Editing in E. coli Genome

The bacterial strains used in this study are listed in Table S3. Dual TAG codons were site-specifically introduced into essential genes *serS*, *murG*, and *dnaA* using the λ-Red recombinase method [16,17,18]. Electrocompetent C321.ΔA exp cells harboring pCas [19,20,21] were induced with 10 mM L-arabinose, transformed with 100 ng pTargetT plasmid and 400 ng donor DNA via electroporation in a pre-chilled 1 mm gap cuvette using a Bio-Rad Gene Pulser II (2.5 kV, 25 μF, 200 Ω) and recovered in SOC medium at 30 °C. Positive clones were selected on LB agar with kanamycin (50 μg/mL) and spectinomycin (50 μg/mL), validated by colony PCR and Sanger sequencing.

### 2.6. Solid Media Escape Frequency

Escape frequency was quantified as the ratio of escape mutant colony-forming units (CFUs) to total viable CFUs according to established protocols [8]. Briefly, cultures were grown under permissive conditions (LB medium supplemented with 1 mM Cl2Y and 100 ng/mL aTc) and harvested in the late exponential phase. Cells were harvested by centrifugation (5000× *g*, 5 min) and washed three times with sterile PBS to remove residual Cl2Y. Then, cells were serially diluted (10-fold gradients) in sterile PBS. Total CFU counts were determined by plating on permissive medium: LB agar containing 1 mM Cl2Y + 100 ng/mL aTc; and non-permissive medium: LB agar without Cl2Y/aTc supplementation. Plates were incubated at 37 °C for 7 days. Escape frequency was calculated as follows:

Escape frequency = CFU_non-permissive_/CFU_permissive_

Data represent mean values from three technical replicates, with error bars indicating ±1 standard deviation [22].

### 2.7. Observation of Bacterial Growth with Suicide Module—Toxin Expression

Bacterial strains engineered to express toxin proteins were cultured in LB medium supplemented with appropriate selection marker antibiotics (kanamycin 50 μg/mL) for 16 h at 37 °C with shaking (250 rpm). Cultures were then diluted 1:100 in fresh LB containing 10 mM L-arabinose to maximize toxin expression induction and incubated for 2 h to allow for toxin accumulation. The bacterial suspension was diluted 10^−5^-fold in solid LB medium and incubated overnight. Bacterial growth dynamics on the solid medium were monitored at 2 h, 4 h, and 12 h post-inoculation under toxin-inducing conditions (10 mM L-arabinose). Morphological changes indicative of toxin activity were documented using an optical microscope (Nikon Inverted Research, Tokyo, Japan).

### 2.8. Quantification and Observation of Bacterial Viability After Cl2Y Removal

Essential gene-based strategy: bacterial cultures were initially propagated in LB medium containing 1 mM Cl2Y with appropriate selection antibiotics for 16 h, followed by supplementation with 1 mM IPTG and 100 ng/mL aTc to induce rescue and Cl2Y module activation. After preconditioning, cells were washed three times with sterile PBS to remove residual Cl2Y. The cell suspensions were then subjected to 100-fold dilution and plated on Cl2Y-depleted agar medium under controlled conditions (37 °C for 12 h).

Toxin–antitoxin-based strategy: Primary bacterial cultures were established in LB medium supplemented with 1 mM Cl2Y and selective antibiotics under standard growth conditions (37 °C, 250 rpm) for 16 h. The cultures also contained 1 mM IPTG and 100 ng/mL aTc to simultaneously activate antitoxin biosynthesis and the Cl2Y expression module. Following preconditioning, cells were harvested by centrifugation (5000× *g*, 5 min) and washed three times with sterile PBS to remove residual Cl2Y. Then, cellular suspensions underwent serial dilutions (10^2^-fold) and were plated on Cl2Y-deficient agar medium enriched with 10 mM L-arabinose to initiate toxin biosynthesis. The plates were then subjected to static incubation under controlled atmospheric conditions (37 °C, for 12 h) to monitor toxin-mediated growth constraints.

### 2.9. Growth Dynamics Quantification of FROs

Temporal progression of bacterial proliferation was monitored through phase-contrast microscopy (Nikon Inverted Research Microscope ECLIPSE Ti2-E/Ti2-E/B) at defined intervals (0, 1, 3, 6, 9, and 12 h), with systematic image acquisition for computational analysis. Generation counts and population distribution patterns were quantitatively evaluated using ImageJ 6.

### 2.10. Statistical Analysis

The reported results represent the averages of at least three biological replicates and two independent biological experiments. GraphPad Prism 8 was used for data processing and graphing, while a two-tailed Student *t*-test was conducted using SPSS software (IBM SPSS Statistics version 25, Tokyo, Japan) for *p*-value testing (two-tailed, two-sample equal variance, * *p*  <  0.05, ** *p*  <  0.01, *** *p*  <  0.001, **** *p*  <  0.0001).

## 3. Results

### 3.1. Design of the Finite-Replicated Organism (FRO)

**Figure 1:**
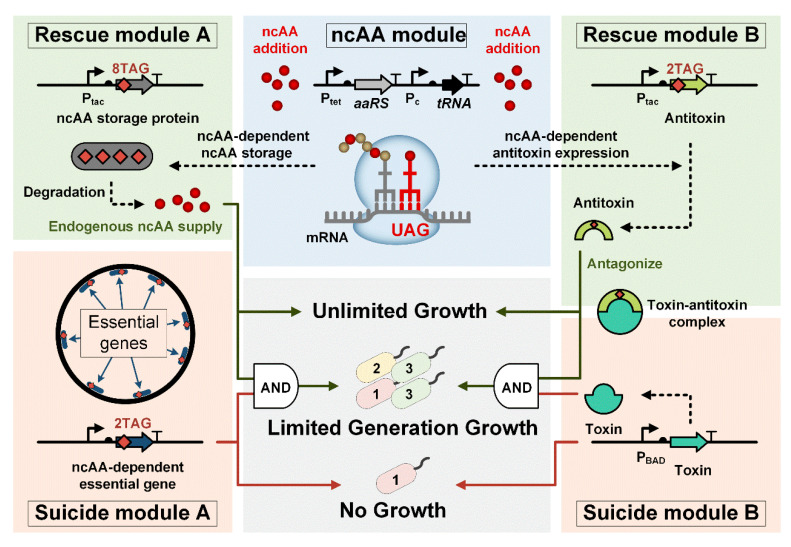
Schematic diagram of the finite-replicated organisms (FROs) framework with three modules: an ncAA module, a rescue module, and a suicide module that releases Cl2Y via controlled proteolysis (degradation arrow), sustaining endogenous ncAA supply for survival. Rescue Module A (dusty green) links ncAA availability to a degradable storage protein gene containing 8 TAG codons. This module is coupled with Ptac-driven expression of essential genes carrying dual TAGs (Suicide Module A, dusty orange), enabling infinite growth only when ncAA-dependent translation machinery (tRNA/aaRS) actively suppresses TAG stop codons. Toxin–Antitoxin (TA) strategy (right pathway, Module B): the same ncAA module drives antitoxin expression (2TAG-coded) to produce antitoxin (Rescue Module B, dusty green). In Suicide Module B (dusty orange), growth hinges on dynamic TA neutralization: antitoxin directly binds toxin (protein interaction node) to permit replication. Finite replication emerges from TAG codon competition (2TAG mRNA vs. translational components), while AND-gate logic triggers irreversible termination upon ncAA depletion or TA imbalance.

An ncAA-dependent FRO consists of three key components: an ncAA module, a suicide module, and a rescue module, which function as the “kill signal”, the “kill switch”, and the “tuner”, respectively. To design the ncAA module, we selected 3,5-dichlorotyrosine (Cl2Y) as the ncAA due to its distinct size and geometry, which offers better specificity compared to other ncAAs [14]. Our preliminary data showed that the Cl2Y OTS exhibited lower leakage and superior translational efficiency than the OTS of p-azido-L-phenylalanine (pAzF) (Figure S1). To implement this system, we constructed a codon-optimized Cl2Y aminoacyl-tRNA synthetase (Cl2YRS) alongside its corresponding tRNA targeting the UAG stop codon [14]. The *E. coli* C321.ΔA exp strain, with all amber codons (TAG) substituted with ochre codons (TAA), was used as a model organism [9] (Figure 1). This system allows TAG sequences to be inserted into *E. coli* genes, enabling the site-specific incorporation of Cl2Y without disrupting normal cellular function [23]. In the absence of Cl2Y, the expression of any *E. coli* gene containing TAG would be prematurely terminated. With the essential gene strategy, an *E. coli* gene critical to normal cellular function was inserted with two TAGs, forming the suicide module (Suicide Module A). A red fluorescent protein (RFP), carrying eight TAGs, was used as the rescue module (Rescue Module A) (Figure 1). Upon the addition of Cl2Y, RFP was expressed with eight Cl2Y incorporated into each molecule. Cl2Y can be released upon the degradation of RFP and acts as the “fuel” for the proper expression of the essential genes, allowing the FRO to survive and grow. With the TA system strategy, we employed a type II toxin as the suicide module (Suicide Module B), while the corresponding antitoxin carrying TAG insertions served as the rescue module (Rescue Module B) (Figure 1). In the presence of Cl2Y, the antitoxin was expressed, neutralizing the toxin and allowing the FRO to continue growing. By adjusting the supply of antitoxin, we were able to control the number of generations the FRO could replicate.

### 3.2. Finite Replication by FROs Based on Essential Gene Strategy

**Figure 2:**
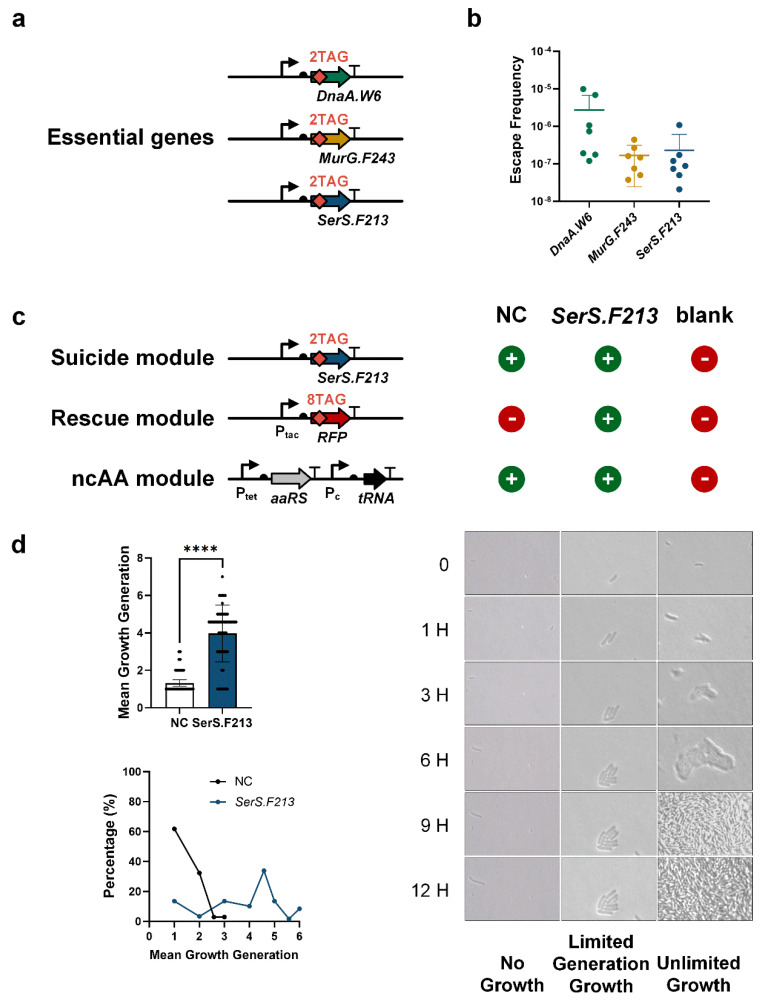
Escape frequency analysis of each suicide module and finite replication dynamics of SerS.F213 FROs. (**a**) **Suicide module design schematic featuring dual TAG insertions in essential genes.** With the gene editing strategy, the *E. coli* essential genes (*dnaA*, *murG*, and *serS*) were inserted with two TAGs, forming the suicide module (Suicide Module A). (**b**) Quantitative escape frequency measurement expressed as CFU ratio ±Cl2Y supplementation (Mean ± SD, N = 7 biological replicates). Escape frequency was quantified as the ratio of escape mutant colony-forming units (CFUs) to total viable CFUs. (**c**) Engineering strategy for SerS.F213 FROs through rescue module insertion. Two plasmids expressing the ncAA orthogonal translation system and storage protein RFP carrying eight TAGs were constructed. When the exogenous supply of ncAA is interrupted, the storage protein RFP with TAGs degrades and releases the expression of genes necessary for ncAA supply. (**d**) The finite replication characteristics of SerS.F213 FROs. Left panel: growth generation quantification (top) and generational distribution (bottom). The SerS.F213-engineered FROs demonstrated finite proliferation, sustaining four generations of growth before growth arrest, while rescue-module-deficient controls (NC) showed unrestricted proliferation (mean ± SD; **** *p* < 0.0001, two-tailed Student *t*-test). Right panel: Microscopic imaging demonstrates finite replication phenotypes in *serS*-modified FRO. The bacterial growth was observed and filmed every 0, 1, 3, 6, 9, and 12 h.

To construct an FRO based on the essential gene strategy, we first selected essential protein candidates using the following criteria: (i) the candidate protein must be crucial for the survival of the organism, and its function should not be compensated for by environmental metabolites [22]. (ii) After incorporating an ncAA, the candidate protein must maintain its normal function without significantly impairing cellular fitness [8,9]. (iii) The selected proteins should be spread across different regions of the genome to reduce the likelihood of escape through a single horizontal gene transfer (HGT) event. Based on these criteria, we selected three *E. coli* essential genes, *dnaA*, *murG*, and *serS*, which are distributed across the genome (Figure S2). DnaA is an initiator involved in chromosomal replication [10]. MurG is an N-acetylglucosaminyltransferase [24], while SerS is a Seryl-tRNA synthetase [25]. Using the λ-Red recombinase method [16,17,18], we introduced dual TAG stop codons into each selected essential gene to construct the suicide modules: SerS.F213, MurG.F243, and DnaA.W6 (Figure 2a), following the previously published ncAA insertion strategy [22] (Figure S2). We then quantified the escape frequency of each suicide module by calculating the ratio of colony-forming units (c.f.u.) on plates with and without Cl2Y (Figure 2b). We observed that the escape frequencies for these suicide modules ranged from 10^−5^ to 10^−7^, with SerS.F213 and MurG.F243 showing substantially lower escape frequencies than DnaA.W6 (Figure 2b). These results were consistent with the previously reported escape frequencies of GROs that had single edited essential genes (approximately 6.7 × 10^−7^) [8,10]. While these escape frequencies were slightly higher than the environmental biosafety standards (<1 × 10^−8^) set by the National Institutes of Health (NIH), we anticipate that the escape frequency could be further reduced by combining multiple ncAA-dependent essential genes or integrating additional suicide mechanisms.

To demonstrate the capability of finite replication, we engineered three distinct FROs using the three suicide modules, incorporating a rescue module and an ncAA module. For each FRO, we also constructed corresponding negative control (NC) samples by pairing the ncAA module with each suicide module (SerS.F213-NC, MurG.F243-NC, and DnaA.W6-NC). Additionally, we created a blank control containing only empty plasmids (Figure 2c and Figure S3). All samples were initially pre-cultured with Cl2Y for 16 h, ensuring that the cells were provided with the necessary “fuel” for growth. Following pre-culture, the cells were cultured on plates lacking Cl2Y and grown for 12 h. We captured the growth of multiple *E. coli* cells at 0, 1, 3, 6, 9, and 12 h to track their proliferation. Our data revealed that most cells from the SerS.F213-based FRO grew for approximately four generations (yielding 16 cells) within 9 h, and stopped proliferating afterwards (Figure 2d, Video S1). In contrast, the SerS.F213-NC cells exhibited minimal to no growth (Video S2), and the blank control cells continued to grow infinitely (Figure 2d, Video S3). After counting all the cells (Figure S4), we discovered that the generations of SerS.F213 FRO cells distributed from one (the first generation) to seven (the seventh generation), which was significantly higher than the SerS.F213-NC cells that mostly remained at the first generation (Figure 2d). It showed that some cells of SerS.F213 FRO (about 10%) could achieve reproductive capabilities for six generations, and these bacteria have undergone a nearly 100-fold expansion (yielding about 64 cells) (Figure 2d and Figure S4). Compared with SerS.F213 FRO, the FROs based on MurG.F243 and DnaA.W6 also demonstrated finite replication, but they exhibited a lower average generation count (Figures S4 and S5). Additionally, by reducing the number of Cl2Y insertions in the rescue module from eight to four, the growth generation of SerS.F213 FRO remained largely the same, indicating that the number of Cl2Y carried in the rescue module could be further reduced (Figure S6). Together, these results demonstrate that FROs can be successfully constructed using the essential gene strategy.

### 3.3. Finite Replication by FROs Based on Type II TA Systems

To further optimize the robustness and growth of the generation, we constructed the FRO based on toxin–antitoxin (TA) systems. We first chose a number of TA pairs following these criteria: (i) both the toxin and antitoxin should be small proteins that belong to type II TA systems, where the antitoxin neutralizes the toxin through direct protein–protein interactions [8,26]. The toxin should target essential cellular processes that are common across a wide range of pathogens, enabling modular application of the system to different species. (iii) The interaction between the toxin and antitoxin should not be the ones with known cross-reactivity to ensure that the antitoxin only neutralizes its corresponding toxin [27,28]. (iv) The selected toxin must efficiently inhibit the growth of *E. coli* upon induction, ensuring a robust “kill switch” mechanism. (v) The antitoxin should only neutralize the toxin and rescue growth when Cl2Y is present, allowing for the regulation of the “kill switch” through ncAA availability. Based on these criteria, we selected five type II TA systems: Doc-Phd, ParDE, CcdAB, Kis-Kid, and Zeta-Epsilon. The five toxins (Doc, ParE, CcdB, Kid, and Zeta) all target critical, universal cellular functions such as protein translation [29], DNA synthesis [30,31], and RNA transcription [32].

**Figure 3:**
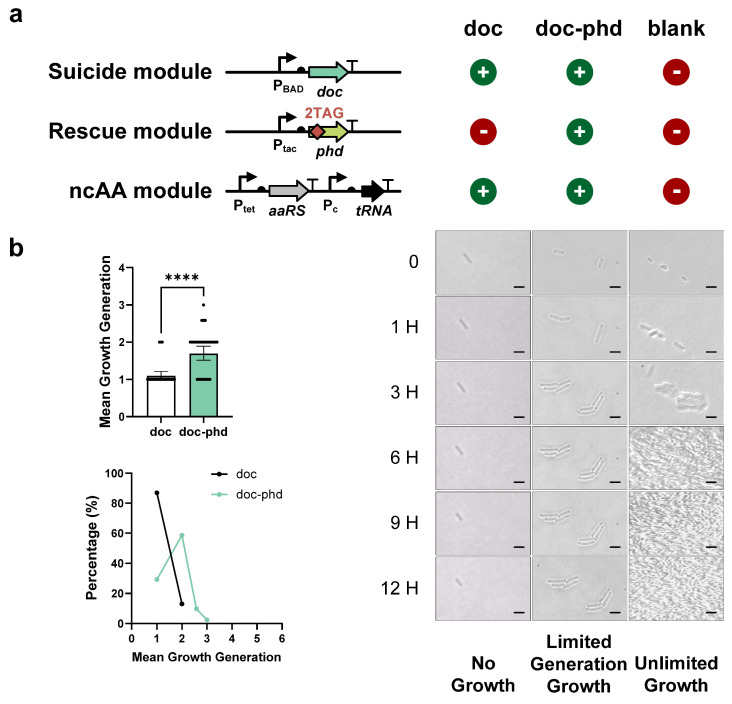
Functional characterization of toxin–antitoxin (TA) engineered FROs. (**a**) **Engineering schematic of Doc-Phd TA system through dual TAG insertion in antitoxin *phd* gene.** The suicide module and ncAA module are assembled on a single plasmid. One plasmid expressing a suicide module - toxin *doc* gene and ncAA module was constructed. Another plasmid expressing rescue module - antitoxin *phd* gene carrying dual TAGs. When the exogenous supply of ncAA is interrupted, the rescue module - antitoxin antagonizes the suicide module - toxin, resulting in toxic inhibition and maintaining growth. (**b**) The finite replication and distribution of Doc-Phd FROs. Left panel: quantitative analysis of the Doc-Phd FROs, including mean growth generations (top) and generational distribution (bottom). The Doc-Phd FRO grew for approximately two generations, while rescue module-deficient controls (Doc) showed unrestricted proliferation (mean ± SD; **** *p* < 0.0001, two-tailed Student *t*-test). Right panel: scale bar represents 2 μm. Microscopic imaging demonstrates finite replication in DocPhd FROs. The bacterial growth was observed and filmed every 0, 1, 3, 6, 9, and 12 h.

To test the killing effect of the toxin, we constructed the suicide modules based on five different toxins (Doc, ParE, CcdB, Kid, and Zeta) with a tightly inducible promoter. The growth of each toxin gene and the blank control was then evaluated using microscopy. Our data demonstrated that only the Doc and ParE toxins exhibited complete inhibition of cell growth, while Kid, Zeta, or CcdB toxins were unable to fully inhibit cell proliferation (Figure S7). Therefore, we proceeded to construct two rescue modules (Phd and ParD antitoxins, respectively) for the Doc and ParE toxin, each of them inserted two TAG codons for site-specific ncAA incorporation. Then, two TA-based FROs were constructed by adding these two rescue modules to their respective toxins. To assess the capability of these FROs for finite replication, all samples were initially pre-cultured with Cl2Y for 16 h, followed by culture on plates without Cl2Y for 12 h. We observed that the Doc-Phd FRO cells proliferated for two generations (yielding four cells) by 3 h and then stopped growing (Figure 3a). The average generation count of all tested Doc-Phd FRO cells was approximately two (Figure 3b and Figure S8), while almost all toxin-Doc cells remained in the first generation (Figure 3b and Figure S8). Moreover, a small number of cells were observed to achieve three generations, and these bacteria yielded about eight cells (Figure 3b and Figure S8). These results demonstrate that ncAA-dependent FROs can be constructed using TA systems.

### 3.4. Modulation of the Growth Generation of FROs

**Figure 4:**
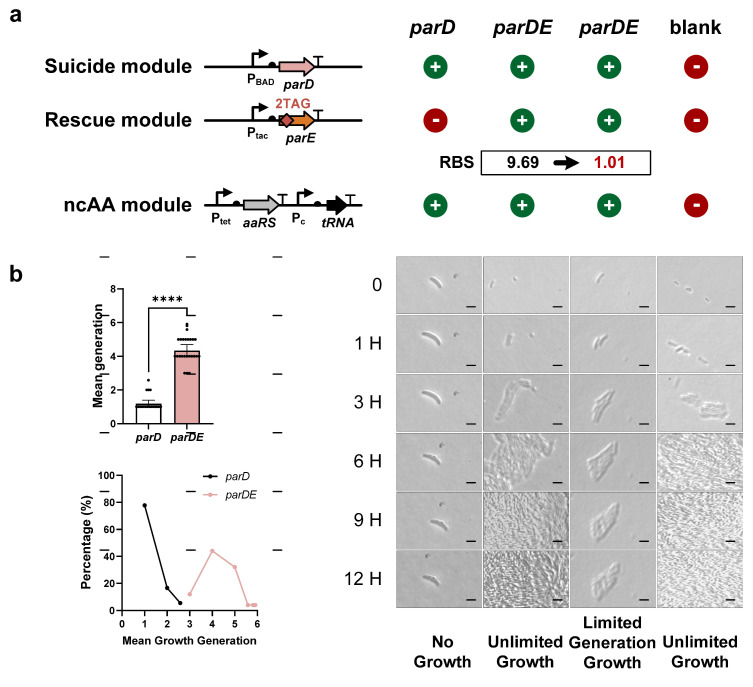
Tunable finite replication in ParDE-engineered FROs. (**a**) **Left panel: schematic of FRO construction through TAG insertion in the *parD* gene of the ParDE system.** Right panel: RBS optimization strategy for *parD* gene (RBS strength reduced from 9.69 to 1.01). The suicide module and ncAA module are assembled on a single plasmid. One plasmid expressing suicide module -toxin *parE* gene and ncAA module was constructed. Another plasmid expressing rescue module- antitoxin *parD* gene carrying dual TAGs. (**b**) The regulation of finite replication analysis in parDE FROs. Left panel: mean generational capacity (top) and generational distribution (bottom). The ParDE-M1 FRO grew for approximately four generations, while ParDE controls showed infinite generation (mean ± SD, **** *p* < 0.0001, two-tailed Student *t*-test). Right panel: scale bar represents 2 μm. Microscopic imaging validation of finite replication in ParDE FROs. The bacterial growth was observed and filmed every 0, 1, 3, 6, 9, and 12 h.

**Figure 5:**
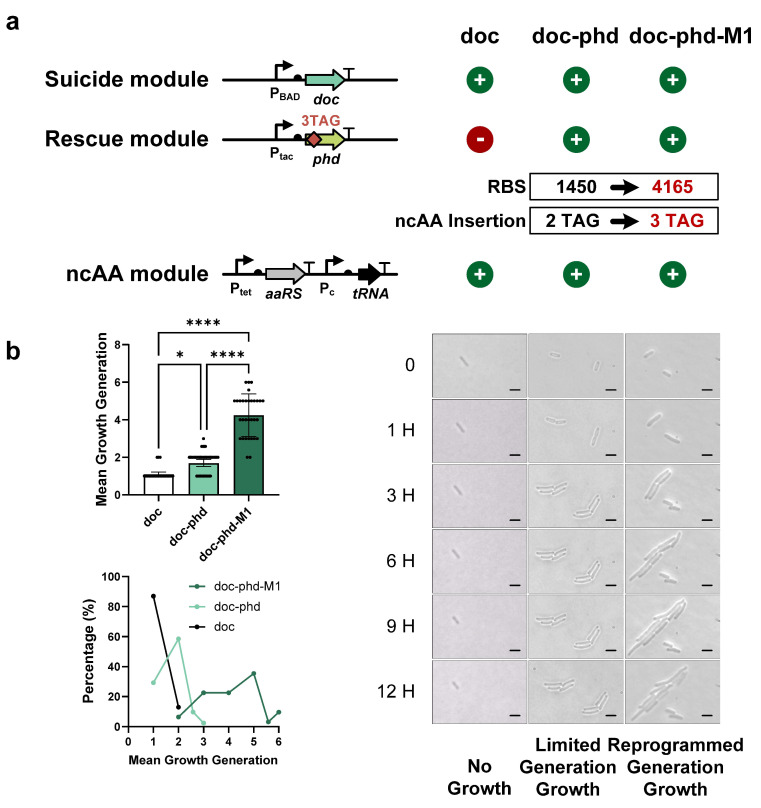
Enhanced dynamic range through RBS optimization and multi-TAG engineering. (**a**) Doc-Phd system engineering schematic: Left panel: schematic diagram illustrating FRO engineering via the Doc-Phd system. Right panel: RBS enhancement of the *phd* gene (RBS strength increased from 1450 to 4165). Leaky expression suppression via TAG-based transcriptional attenuation via additional TAG insertion in the *phd* gene, resulting in the construction of Doc-Phd-M1 FROs. (**b**) Comparative growth analysis: the mean growth generations (top) and distribution (bottom) of Doc-Phd-M1 FROs. Doc-Phd-M1 engineered FROs demonstrated enhanced reproductive control, sustaining four generations of proliferation compared to the parental Doc-Phd strain (2 generations) (mean ± SD; **** *p* < 0.0001, two-tailed Student *t*-test). Right panel: scale bar represents 2 μm. Microscopic visualization of improved growth restriction in Doc-Phd-M1 FROs. The bacterial growth was observed and filmed every 0, 1, 3, 6, 9, and 12 h.

To improve the number of generations of FROs, we optimized the supply of antitoxins through two approaches: adjusting RBS strength and modifying the number of ncAA insertions. First, we constructed a second TA-based FRO using the ParDE system. Unlike the Doc-Phd FRO, the initial ParDE FRO exhibited infinite cell proliferation rather than finite replication (Figure 4a), likely due to excess antitoxin expression. Using the RBS calculator, we designed a new RBS sequence for the ParD antitoxin, reducing its strength from 9.69 to 1.01, and created the modified ParDE-M1 FRO (Figure 4a). Our evaluation showed that ParDE-M1 FRO cells grew between three and six generations, with an average generation count of approximately four (yielding 16 cells) (Figure 4b and Figure S8), indicating that finite replication of FROs could be achieved by reducing antitoxin RBS strength. We also discovered that nearly half of the ParDE-M1 FRO cells (about 40%) could achieve reproductive capabilities for five to six generations, and these bacteria have undergone a nearly 100-fold expansion (Figure 4b and Figure S8). Next, we extended the dynamic range of the Doc-Phd FRO by increasing the RBS strength for the Phd antitoxin and adding more TAGs within the *phd* gene. In TA-based FROs, the number of generations tends to increase with higher antitoxin expression levels. However, enhancing the RBS strength of *phd* from 1450 to 4165 resulted in Cl2Y-independent leakage expression, causing infinite proliferation (Figure 5a). To counter this leakage, we inserted an additional TAG into the *phd* gene, creating the Doc-Phd-M1 FRO. Under the same evaluation protocol, Doc-Phd-M1 FRO achieved finite replication growth with a higher average generation count, which was approximately four generations (yielding 16 cells) compared to the original Doc-Phd FRO (Figure 5b and Figure S8). Compared with the Doc-Phd FRO, which could only achieve the highest reproduction of three generations, the optimized Doc-Phd-M1 FRO was able to achieve up to six generations, and the bacterial amplification was increased by nearly 100 times (Figure 5b). These results demonstrated that a higher number of generations of FROs can be achieved by enhancing the RBS strength of antitoxins, and additional ncAA insertions were necessary to prevent ncAA-independent growth. In summary, we demonstrated the ability to control FRO growth by modulating the supply of antitoxins. This approach could be applied to develop new FROs or regulate the growth of existing FROs effectively.

## 4. Discussion and Conclusions

Recently, the WHO published a list of globally prioritized endemic pathogens that require urgently developed new vaccines. Among these are various bacteria, notably *Staphylococcus aureus* and *Klebsiella pneumoniae*. Additionally, bacteria such as *Pseudomonas aeruginosa* and *Acinetobacter baumannii* have long been linked to instances of hospital-acquired infections (HAIs). These pathogens have evolved strains that are resistant to multiple antibiotics, presenting a significant threat to human health, particularly affecting immunocompromised individuals and hospitalized patients. With the growing severity of antimicrobial resistance (AMR), vaccines stand out as the most effective antibiotic-free strategy for controlling and preventing the spread of these pathogens. Live-attenuated vaccines are a common type of vaccine for bacterial pathogens, including those against measles, mumps, and rubella [33]. Nevertheless, owing to their replicative potential, live-attenuated vaccines inherently carry the risk of infecting vaccine recipients. To mitigate this risk, subunit vaccines employed the strategy of selecting only essential antigens for vaccine development. This strategy requires a comprehensive understanding of the pathogens’ mechanisms of infection to identify effective antigens and to intricately engineer vaccines for optimal immunogenicity. This task can be particularly challenging for pathogens with a large array of antigens. Alternatively, the safety of live-attenuated vaccines can be improved by preventing infinite growth after vaccination.

In this study, we present an FRO framework that restricts the growth of live-attenuated vaccines to a finite replication, thereby reducing the risk of infection. Importantly, FRO-based vaccines achieve up to 100-fold bacterial amplification (about six generations) through controlled proliferation cycles, producing substantially greater antigen payloads that drive stronger immune activation than conventional inactivated vaccines. Notably, we provided a systematic approach to programming the number of generations, which allows FRO-based vaccines to achieve balanced safety and immunogenicity. Specifically, to program the growth of FROs, two distinct strategies were employed: the essential gene strategy and the toxin–antitoxin (TA) strategy. The essential gene strategy links fundamental biological functions, like DNA replication and RNA transcription, to the presence of ncAA, thereby effectively managing escape frequencies and ensuring the safety of FROs. Conversely, the TA strategy employs the highly specific interaction between paired toxin and antitoxin to modulate the growth of FROs, which offers the capability of fine-tuning the dynamic range of FROs’ generation count. The extensive variety of TA systems found in nature presents significant potential for the excavation of TA systems specifically for FROs. Both systems operate by making the growth of FRO dependent on a single factor, the amount of the same ncAA (Cl2Y), which makes them compatible for integration into the same FRO with both low escape frequency and high programmability. This study not only presents an innovative approach for vaccine development through the use of FROs but also opens new avenues for applications in the field of metabolic engineering. By leveraging synthetic biology strategies, particularly the use of ncAA systems [34], we can precisely modulate bacterial metabolic pathways to enhance vaccine safety and efficacy. Furthermore, the design of FROs allows for the controlled synthesis of metabolic products, thereby expanding their application in industrial biotechnology, such as enabling controllable fermentation processes for the production of pharmaceuticals or bio-based chemicals.

Our study still has some limitations. Using *E. coli* as the model organism, we demonstrated a proof-of-concept framework of engineered FROs, which was not as clinically relevant as pathogens such as *S. aureus* or *P. aeruginosa*. Thus, in this study, the immune responses of FROs as vaccines were not evaluated. Meanwhile, FROs were demonstrated using only one essential gene and achieved comparable escape frequency as previous studies. For the next step, multiple essential genes should be included into FROs to ensure the escape frequency meets the standard (<1 × 10^−8^) set by the NIH in short- and long-term cultures, and FRO-based live-attenuated vaccines should be constructed by transferring FROs to clinically relevant pathogens to evaluate the safety, immunogenicity, and protective efficacy.

In summary, we provided a programmable FRO framework to make live-attenuated vaccines safer while maintaining high immunogenicity. We believe this work represents an innovative application of synthetic biology in the field of vaccinology and would contribute to the global effort to combat antimicrobial resistance. Future research could focus on employing systems biology tools to further optimize the metabolic networks of FROs, addressing potential metabolic stress issues while enhancing yield and efficiency. Through engineering, our findings not only ensure the safety of vaccines but also provide new perspectives for the application of metabolic engineering in synthetic biology, thereby advancing related technologies in industrial applications.

## Data Availability

Any additional data that support the findings of this study are available from the corresponding author upon request. All plasmids and strains used in this study are available from the corresponding author under a material transfer agreement.

## Supplementary Materials

The following supporting information can be downloaded at https://www.mdpi.com/article/10.3390/life15091381/s1: Table S1: Plasmids used in this article; Table S2: The RBS fragment used in this article; Table S3: Bacteria Strains used in this article; Table S4: Essential genes used in this study; Table S5: Escape frequencies of FROs based on essential genes strategy; Table S6: Evaluation of ncAA systems based on pAzF and Cl2Y; Table S7: Mean growth quantification of FROs based on essential genes strategy; Table S8: Mean growth number of SerS.F243 FROs engineered with 4-TAG versus 8-TAG codon insertions; Table S9: Mean growth generation of FROs based on type II TA systems; Figure S1. Orthogonal ncAA system screening and performance characterization; Figure S2. The position and mutation of essential genes on the *E. coli* genome; Figure S3. Design of FROs based on TAG insertions in essential genes *serS* (a), *murG* (b) and *dnaA* (c); Figure S4. Typical microscopy of finite replication of FROs based on essential gene strategy. The TAG insertions in essential genes *serS* (a), *murG* (b) and *dnaA* (c); Figure S5. The finite replication analysis and distribution of DnaA.W6 and MurG.F246 FROs; Figure S6. Functional validation of TAG codon optimization in SerS.F213 FRO rescue modules; Figure S7. Screening of toxin-mediated suicide modules; Figure S8. Representative microscopy of finite replication of FROs based on TA systems through TAG insertions in antitoxin *phd* (a) and *parD* (b); Video S1: Time-lapse observation of the typical single colony in the SerS.F213 FRO; Video S2: Time-lapse observation of a single colony in the SerS.F213-NC sample; Video S3: Time-lapse observation of the typical single colony in the blank control cells.
