# Supplementary material for: A Programmable Finite-Replicated Organism Framework for Balanced Safety and Functionality
Source: Life (Basel). 2025 Sep 1;15(9):1381. doi: 10.3390/life15091381 (PMC12471649; doi:10.3390/life15091381)
Supplement: Supplementary file 1 [file life-15-01381-s001.zip › life-3769233-supplementary.pdf]

Supplementary information for

A programmable finite-replicated organism framework for balanced safety and functionality

Authors:

Mengyuan Wang [1], Pei Du [2], Fankang Meng [2], Wenhui Zhang [3], Yanhui Xiang [3,*], Qiong Wu [1,*] and Chunbo Lou [3,*]

[1] State Key Laboratory of Green Biomanufacturing, MOE Key Lab. Bioinformatics, Center for Synthetic and Systems Biology, School of Life Sciences, Tsinghua University, Beijing 100084, China; wangmy5600@163.com

[2] CAS Key Laboratory of Pathogen Microbiology and Immunology, Institute of Microbiology,
Chinese Academy of Sciences, Beijing 100101, China; dupeicn@gmail.com (P.D.);
mengfankang1994@gmail.com (F.M.)

[3] Center for Cell and Gene Circuit Design, State Key Laboratory of Quantitative Synthetic Biology, Shenzhen Institute of Synthetic Biology, Shenzhen Institutes of Advanced Technology, Chinese Academy of Sciences, Shenzhen 518055, China; zhangwenhui1994@126.com

* Correspondence: yh.xiang@siat.ac.cn (Y.X.); wuqiong@mail.tsinghua.edu.cn (Q.W.); louchunbo@gmail.com (C.L.)

Table S1: Plasmids used in this article

Plasmid name	Information	Function	First occurrence site
GFP-2TAG	lac promoter-GFP (2 TAG coden was inserted after the start codon)	Report the transfer efficiency of ncAA system	Fig S1
pTet-pAzF OTS	pTet-pAzF aaRS- Prok-tRNA	pAzF OTS which produces pAzF aminoacyl-tRNA synthetase induced by aTc (Mandell et al., 2015)	Fig S1
pTet-Cl2Y OTS	pTet-Cl2Y aaRS- Prok-tRNA	Cl2Y orthogonal translation system (Liu et al., 2014) which produces pAzF aminoacyl-tRNA synthetase induced by aTc	Fig S1
pCas	Pcas-cas9-pBAD-gam-beta-exonuclease-Ptrc-sgRNA-PMB1	Expressing Cas9 protein and λ-Red recombinase system (Gam, beta, and exonuclease) and producing gRNA that targeted pMB1 ori in ptargetF plasmid (Jiang et al., 2015)	Fig S2
ptargetF-dnaA-F6	pJ23119-dnaa F6-gRNA	Producing gRNA of *dnaA* gene and cas9 binding site	Fig S2
ptargetF-murG F243	pJ23119- murG F243-gRNA	Producing gRNA of *murG* gene and Cas9 binding site	Fig S2
ptargetF-serS F213	pJ23119- serS F213-gRNA	Producing gRNA of *serS* gene and cas9 binding site	Fig S2
pTac-RFP	pTac-storage protein gene: RFP without TAG coden	Expressing storage protein-RFP without TAG codons as Negative Control	Fig 2

pTac-RFP-4TAG	pTac-storage protein gene: RFP (4 TAG coden was inserted after the start codon	Expressing storage protein-RFP holding four TAG codons induced by IPTG	Fig S3
pTac-RFP-8TAG	pTac-storage protein gene: RFP (8 TAG coden was inserted after the start codon)	Expressing storage protein-RFP holding eight TAG codons induced by IPTG	Fig 2
pBAD-Doc	pBAD-Doc gene	Expressing toxin-Doc induced by arabinose	Fig S7
pBAD-ParE	pBAD-ParE gene	Expressing toxin-ParE induced by arabinose	Fig S7
pBAD-CcdB	pBAD-CcdB gene	Expressing toxin-CcdB induced by arabinose	Fig S7
pBAD-Kid	pBAD-Kid gene	Expressing toxin-Kid induced by arabinose	Fig S7
pBAD-Zeta	pBAD-Zeta gene	Expressing toxin-Zeta induced by arabinose	Fig S7
pTac-Phd-2TAG-1450	pTac-Phd gene (2 TAG coden was inserted after the start codon)	Expressing antitoxin-Phd controlled by Cl2Y coden- TAG (RBS=1450)	Fig 3
pBAD-Doc-pTet-Cl2Y OTS	pBAD-Doc gene& pTet-Cl2Y aaRS- Prok-tRNA	Expressing toxin-Doc and Cl2Y orthogonal translation system (Liu et al., 2014)	Fig 3
pTac-parD-2TAG-9.69	pTac-parD gene (2 TAG coden was inserted after the start codon)	Expressing antitoxin-parD controlled by Cl2Y coden- TAG (RBS=9.69)	Fig 4
pTac-parD-2TAG-1.01	pTac-parD gene (2 TAG coden was inserted after the start codon)	Expressing antitoxin-parD controlled by Cl2Y coden- TAG (RBS=1.04)	Fig 4
pTac-Phd-3TAG-4165	pTac-Phd gene (3 TAG coden was inserted after the start codon)	Expressing antitoxin-Phd controlled by Cl2Y coden- TAG (RBS=4165)	Fig 5

List of all the plasmid vectors engineered for the finite-replicated organism(FRO) framework development.

Table S2: The RBS fragment used in this article

RBS name	RBS strength	RBS sequence
Phd-1	1450	AAAAACTAGAGACTAGTCTTTAAGAGAATACCA
Phd-2	4165	AAAAACTAGAGACTAGTCTTTTTAAGAAGGAGATATACAT
parD-1	9.69	AAAAACTAGAGACTAGTCTTTAAGAGAATACCA
parD-2	1.01	AAAAACTAGAGACTAGTCTTTAAGAGAATACCACACTTGCCACACATCTTAACAG

RBS strength values were predicted using the RBS Calculator (Salis, 2011).

Table S3: Bacteria Strains used in this article

Strain	Genotype	Source
E.coli C321.deltaA.exp (Lajoie MJ, 2013)	*E. coli* MG1655 Delta(ybhB-bioAB)::zeoR Delta prfA; all 321 UAG codons changed to UAA	Addgene #87359
C321.deltaA.exp:: dnaA W6	*E.coli* C321.deltaA.exp（dnaA F6 was replaced by two TAG code）	Edited by λ-Red recombinase method
C321.deltaA.exp::murG F243	*E.coli* C321.deltaA.exp（murG F243 was replaced by one TAG code）	Edited by λ-Red recombinase method
C321.deltaA.exp::serS F213	*E.coli* C321.deltaA.exp（serS F213 was replaced by two TAG code）	Edited by λ-Red recombinase method

List of all the *E. coli* strains and genetic modifications including TAG codon insertions in essential genes (*dnaA/murG/serS*).

Table S4: Essential genes used in this study

Gene	Description	Position of edited loci[a]	Source
serS	Arginyl-tRNA synthetase critical for translation fidelity	F213 locus	(Galindo-Feria et al., 2022)
murG	Undecaprenyl-phosphate glycosyltransferase for peptidoglycan biosynthesis	F243 locus	(Ha et al., 2001)
dnaA	Replication initiation factor controlling chromosome duplication	W6 locus	(Abe et al., 2007)

The essential genes harboring TAG codons in synthetic auxotrophs from this study and associated description of their functions.
Position of edited loci are abbreviated as follows: Phenylalanine (F), Tryptophan (W), and the numbers represent the positions of the corresponding amino acids that have been replaced by Cl2Y.

Table S5: Escape frequencies of FROs based on essential genes strategy

Strain	Escape rate (escapees/c.f.u.)						
DnaA.W6	1.07E-06	1.2E-07	1.76E-07	6.9E-06	7.4E-07	9.9E-06	1.91E-07
MurG.F243	4.4E-07	1.47E-07	3.75E-08	7.5E-08	2.66E-07	1.61E-07	5E-08
SerS.F213	5E-08	1.74E-07	1.2E-07	8.8E-08	7.39E-08	1.08E-06	2.1E-08

List of representative escape rates illustrated in Fig. 2b. The table quantifies escape rates at day 7 for FRO strains engineered with dual amber (TAG) stop codons in essential genes (*serS*, *murG*, *dnaA*). Each column represents an independent technical replicate (n=7), with values reported as escape frequency ± s.d. Escape frequencies were calculated as the ratio of colony-forming units (CFU) on non-permissive media (lacking Cl2Y and induction supplements) to CFU on permissive media (supplemented with 1 mM Cl2Y and 100 ng/mL aTc), following protocols detailed in the Methods (Section 2.6).

Table S6: Evaluation of ncAA systems based on pAzF and Cl2Y.

aTc (ng/ml)	2TAG + Cl2Y			2TAG + Cl2Y + 1mM ncAA			2TAG + pAzF			2TAG + pAzF + 1mM ncAA			Negative Control		
0	1.46E+02	1.41E+02	1.35E+02	2.64E+03	2.62E+03	2.41E+03	3.17E+02	2.92E+02	2.70E+02	1.09E+04	1.11E+04	1.07E+04	9.89E+01	9.53E+01	7.54E+01
5E-05	1.59E+02	1.53E+02	1.46E+02	2.76E+03	2.76E+03	2.44E+03	3.40E+02	3.24E+02	3.06E+02	1.08E+04	1.10E+04	1.11E+04	1.11E+02	1.02E+02	8.11E+01
5E-04	1.69E+02	1.61E+02	1.54E+02	2.70E+03	2.72E+03	2.49E+03	3.83E+02	3.47E+02	3.26E+02	1.11E+04	1.09E+04	1.06E+04	1.14E+02	1.06E+02	8.51E+01
5E-03	1.77E+02	1.68E+02	1.58E+02	2.77E+03	2.83E+03	2.57E+03	3.89E+02	3.70E+02	3.53E+02	9.94E+03	1.09E+04	1.05E+04	1.46E+02	1.11E+02	8.56E+01
5E-02	1.83E+02	1.74E+02	1.64E+02	4.67E+03	4.51E+03	3.94E+03	4.48E+02	4.16E+02	4.01E+02	1.28E+04	1.44E+04	1.38E+04	1.36E+02	1.14E+02	9.04E+01
5E-01	1.89E+02	1.76E+02	1.67E+02	1.09E+04	1.05E+04	1.04E+04	9.60E+02	9.31E+02	9.54E+02	2.09E+04	2.16E+04	2.12E+04	1.39E+02	1.13E+02	8.69E+01
5	2.07E+02	1.94E+02	1.85E+02	1.30E+04	1.25E+04	1.17E+04	4.27E+03	4.21E+03	4.08E+03	2.08E+04	2.12E+04	2.09E+04	1.53E+02	1.16E+02	9.44E+01
50	2.65E+02	2.57E+02	2.33E+02	1.45E+04	1.35E+04	1.25E+04	6.83E+03	6.50E+03	6.34E+03	1.90E+04	2.00E+04	1.84E+04	2.43E+02	9.30E+01	8.22E+01

Flow cytometry-measured GFP fluorescence intensity reflecting ncAA incorporation fidelity. Experimental conditions defined by: TAG codon number in GFP reporter (2TAG), ncAA type (Cl2Y or pAzF), ncAA supplementation (±1 mM). n=3 technical replicates, ±s.d.

Table S7: Mean growth quantification of FROs based on essential genes strategy

serS		*dnaA*		*murG*	
NC	SerS.F213	NC	DnaA.W6	NC	MurG.F243
1	2	1	1	1	1
1	3	2	1	2	4
1	3	4	2	4	4
1	3	2	2	2	1
1	3	2	2	2	1
1	3	2	2	2	1
1	4	1	2	1	1
2	4	1	2	1	1
2	4	1	2	1	1
2	4	1	4	1	2
2	4	1	4	1	2
3	4	3	4	3	2
3	4	1	8	1	2
	4	1	1	1	2
	4	1	1	1	2
	4	1	2	1	2
	5	1	2	1	2
	5	1	2	1	2
	5	1	2	1	1
	5	1	2	1	1
	5	1	2	1	1
	5	1	4	1	1
	5	1	4	1	1
	5	1	4	1	1
	5	1	4	1	1
	5	1	4	1	1
	5	1	4	1	1
	5	1		2	1
	5	1		2	1
	6	1		2	1
	6	2		4	1
	6	2		1	2
	6	2		1	2
	6	2		1	2
	6	3		1	2
	7	4		2	2

3	2
4	2
	2
	2
	2
	2
	2
	2
	2
	2
	4
	4
	4
	4
	4
	4
	4
	4
	4
	4
	4
	4
	4
	4
	4
	4
	4
	4
	8
	8
	8
	8
	8
	8

List of representative growth quantification of FROs illustrated in Fig. 2d.The table quantifies the progeny count per single clone following 12-hour incubation in non-permissive media (Cl2Y-depleted). Negative control (NC) strains without rescue modules and FROs with dual-TAG mutations in essential genes (serS.F213, dnaA.W6, murG.F243). Quantified via time-lapse microscopy

Table S8: Mean growth number of SerS.F243 FROs engineered with 4-TAG versus 8-TAG codon insertions

NC	SerS-4TAG	SerS-8TAG
1	2	1
1	2	1
1	2	1
1	4	1
1	4	1
1	4	1
1	4	1
1	8	1
1	8	2
1	8	2
1	12	4
1	12	4
1	12	4
1	12	4
1	16	4
1	16	4
1	16	4
1	16	4
1	16	8
1	24	8
1	24	8
2		8
2		8
2		8
2		12
2		12
2		12
2		12
2		12
2		12
2		12
2		12
3		12
4		12
		12
		12
		12

12
12
12
12
12
12
12
16
16
16
16
16
16
16
16
24
32
32
32
32
32
64

The offspring count per single clone after 12-hour incubation under non-permissive conditions (Cl2Y-depleted media). SerS-4TAG: Rescue module expressing RFP with 4 TAG codons, SerS-8TAG: Rescue module expressing RFP with 8 TAG codons and Control group (NC): Rescue module-deficient strain. Proliferation quantified via phase-contrast microscopy at 12 h.

Table S9: Mean growth generation of FROs based on type II TA systems

Doc	Doc-Phd	Doc-Phd-M1	ParE	ParDE-M1
1	1	2	1	3
1	1	2	1	3
1	1	3	1	3
1	1	3	1	4
1	1	3	1	4
1	1	3	1	4
1	2	3	1	4
1	2	3	1	4
1	2	3	1	4
2	2	4	1	4
2	2	4	1	4
1	2	4	1	4
1	2	4	1	4
1	2	4	1	4
1	2	4	2	5
1	2	4	2	5
1	2	5	2	5
1	2	5	3	5
1	2	5		5
1	2	5		5
1	3	5		5
1	3	5		5
2	3	5		6
	3	5		6
	1	5		6
	1	5		
	1	5		
	1	6		
	1	6		
	1	6		
	2	6		
	2			
	2			
	2			
	2			
	2			

2
2
2
2
3

List of representative growth quantification of FROs illustrated in Fig. 4b, 5b. The reproductive output of individual clones following overnight culture (12 h) in Cl2Y-free medium are documented. Columns represent engineered TA systems: Doc, Doc-Phd, Doc-Phd-M1 (optimized RBS + 3TAG) and ParE, ParDE-M1 (RBS-tuned variant). Proliferation quantified via phase-contrast microscopy at 12 h.

Supplementary Figures

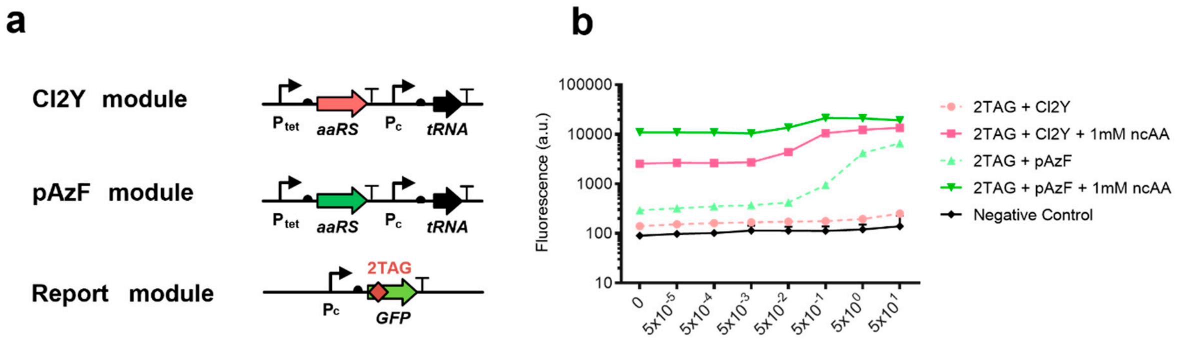

Figure S1. Orthogonal ncAA system screening and performance characterization.

(a) Modular architecture of orthogonal translation systems. The ncAA module comprises a pTet promoter-driven cassette encoding aaRS and cognate tRNA for ncAA incorporation at TAG codons. Both systems feed into a GFP reporter containing dual TAG codons (2TAG) for functional validation. (b) Evaluation of ncAA systems based on pAzF and Cl2Y. Cl2Y system (green) showed baseline activation (1.2×10^4 a.u.) without ncAA, increasing 8.3-fold with 1 mM ncAA (****$P < 0.0001$ vs. negative control), indicating leaky but regulatable TAG suppression. Negative controls (gray) confirmed system specificity. Data normalized to OD600 (mean ± SD, N=3 biological replicates; two-tailed Student's t-test).

a

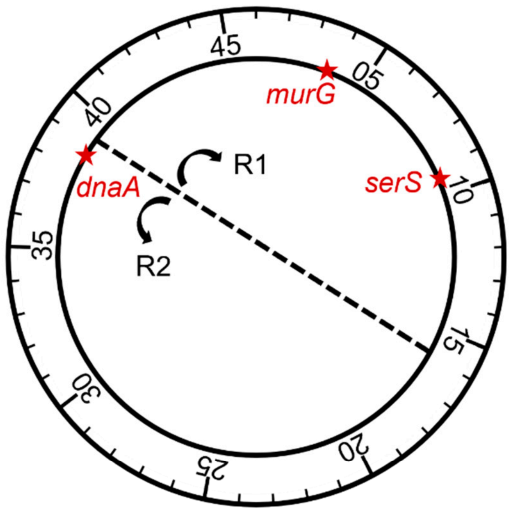

b

>serS

CAACTGCCGAAATTTGCTGGCGATCTGTTCCATAC
TCGTCCGCTGGAAGAA

>serS.F213

CAACTGCCGAAG**TAGTAG**GCAGATCTGTTCCATA
CTCGTCCGCTGGAAGAA

>murG

AAAGTGACGGAATTTATTGATGATATGGCGGCGGC
GTATGCGTGGGCGGAT

>murG.F243

AAAGTGACGGAA**TAGTAG**ATCGACGACATGGCGG
CGGCGTATGCGTGGGCGGAT

>dnaA

GTGTCACTTTCGCTTTGGCAGCAGTGTCTTGCCCG
ATTGCAGGATGAGAG

>dnaA.W6

GTGTCGCTGTCAATT**TAGTAG**CAGCAGTGTCTTGC
CCGATTGCAGGATGAGAG

Figure S2. The position and mutation of essential genes on the *E. coli* genome.

(a) Chromosomal mapping of genomic engineering of essential genes with 2TAG mutations. The circular *E. coli* genome map highlights three engineered loci (red pentagons). Dashed grey lines demarcate replication timing zones R1 (early) and R2 (mid-phase). (b) Precision editing of 2TAG stop codons. Sequence alignments show site-directed insertion of dual in-frame TAG codons (red).

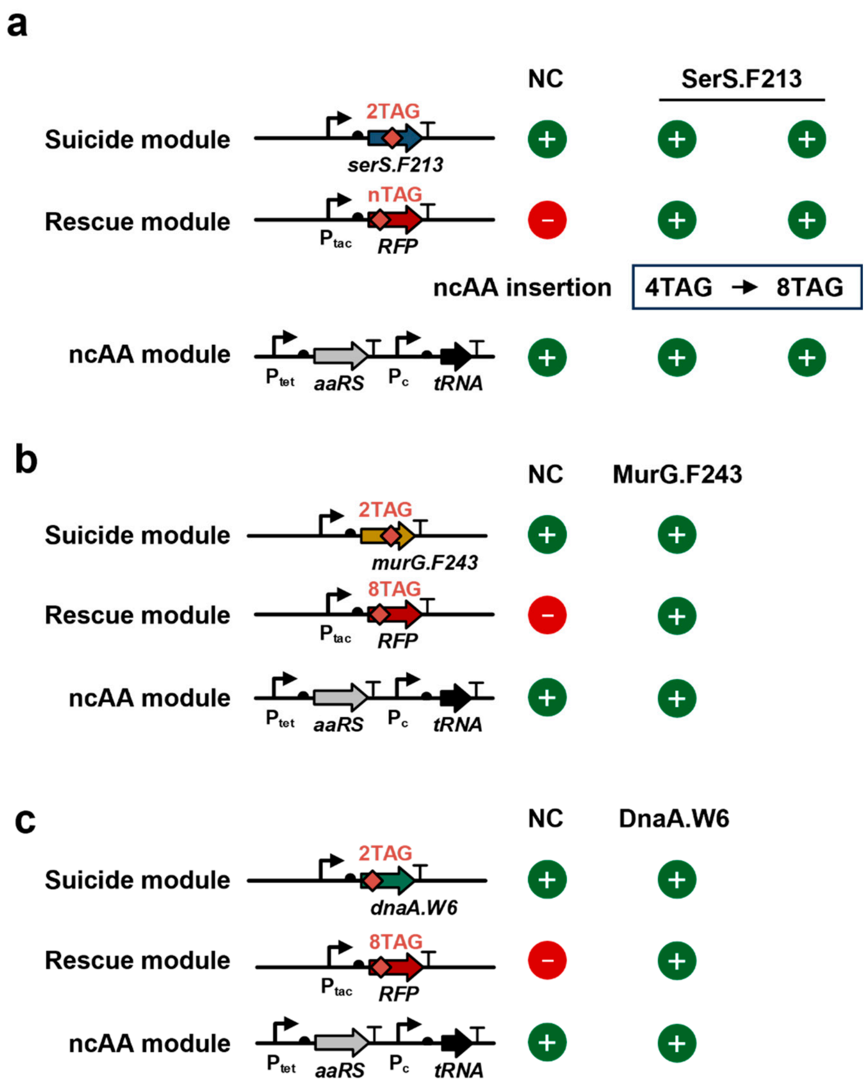

Figure S3. Design of FROs based on TAG insertions in essential genes *serS* (a), *murG* (b) and *dnaA* (c).

Left: Three-module design for FRO. Right: Experimental design of growth dynamics. (a) SerS.F213-based FRO architecture: Suicide module: Chromosomal *serS* gene with 2TAG codons at F213 locus disrupts arginyl-tRNA synthetase function; Rescue module: pTac-driven suppressor tRNA and aaRS (nTAG system) with co-expressed RFP (4TAG/8TAG variants shown); ncAA module (blue): pTet-regulated Cl2Y storage protein (8TAG) + constitutive tRNA expression (Pc promoter). Right: NC (no rescue module), SerS.F213 + 4TAG rescue, and SerS.F213 + 8TAG rescue. (b) MurG.F243 FRO system. Suicide module: 2TAG-mutated *murG* at F243; (c) DnaA.W6 replication-origin FRO. Suicide module: Dual TAG codons in *dnaA*.

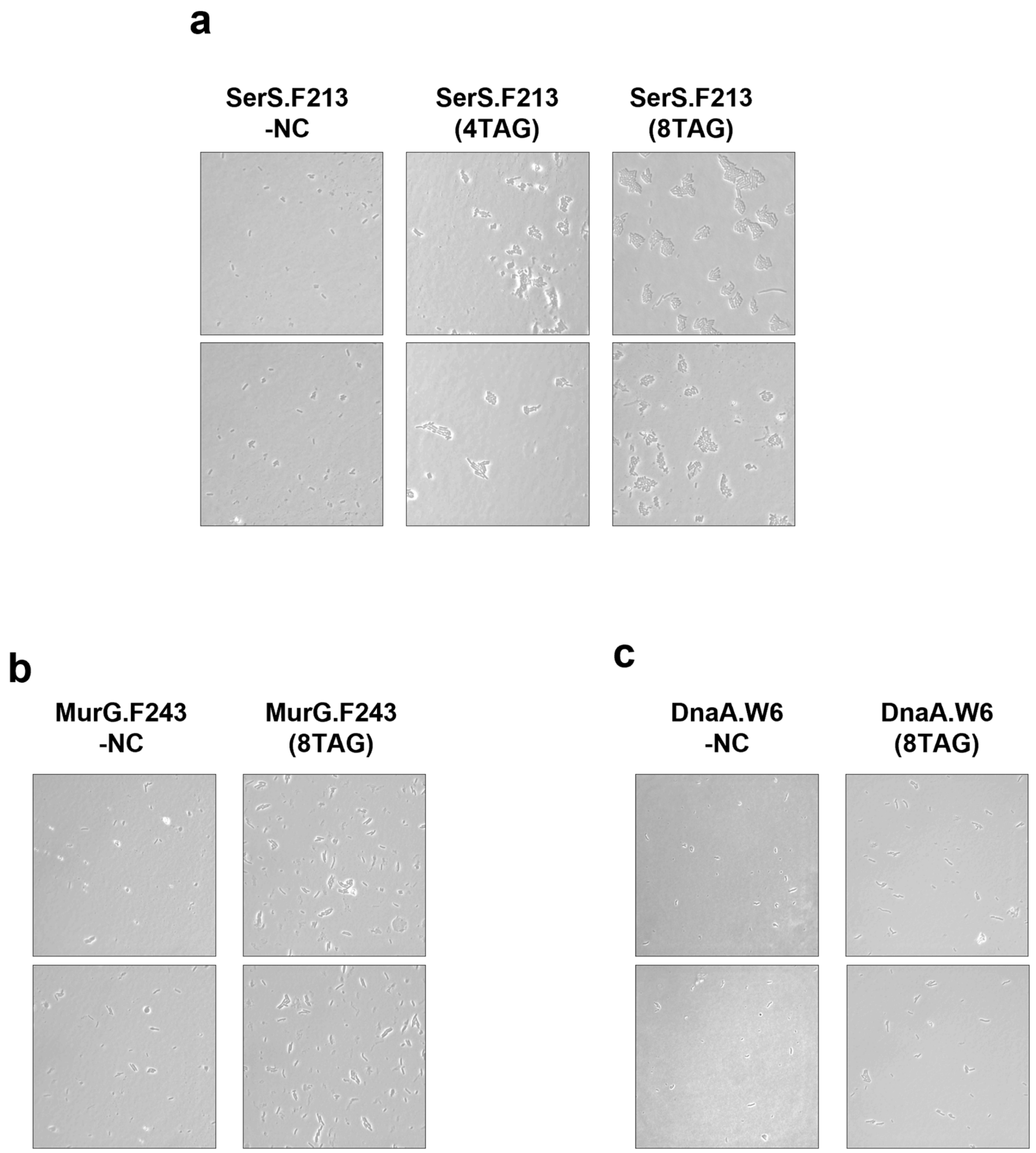

Figure S4. Typical microscopy of finite replication of FROs based on essential gene strategy. The TAG insertions in essential genes *serS* (a), *murG* (b) and *dnaA* (c).

Finite replication phenotypes are observed and imaged by the optical microscope (Nikon Inverted Research Microscope ECLIPSE Ti2-E/Ti2-E/B). The bacterial growth was observed and filmed at 12 hours.

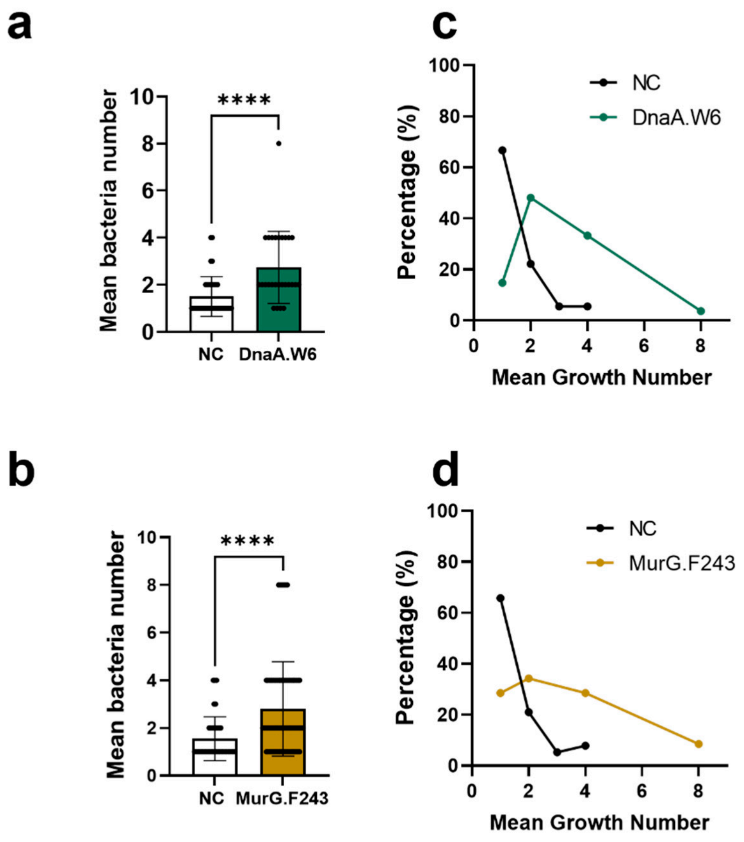

Figure S5. The finite replication analysis and distribution of DnaA.W6 and MurG.F246 FROs.

(a and b) The mean growth numbers of DnaA.W6 (a) and MurG.F246 (b). The Dnaa.W6 and MurG.F243 FROs both demonstrated finite replication, sustaining two generations of growth before growth arrest, while rescue module-deficient controls (NC) showed unrestricted proliferation (mean ± SD, ****$P <$ 0.0001, two-tailed Student's t-test). (c and d) The distribution of numbers of DnaA.W6 (c) and MurG.F246 (d).

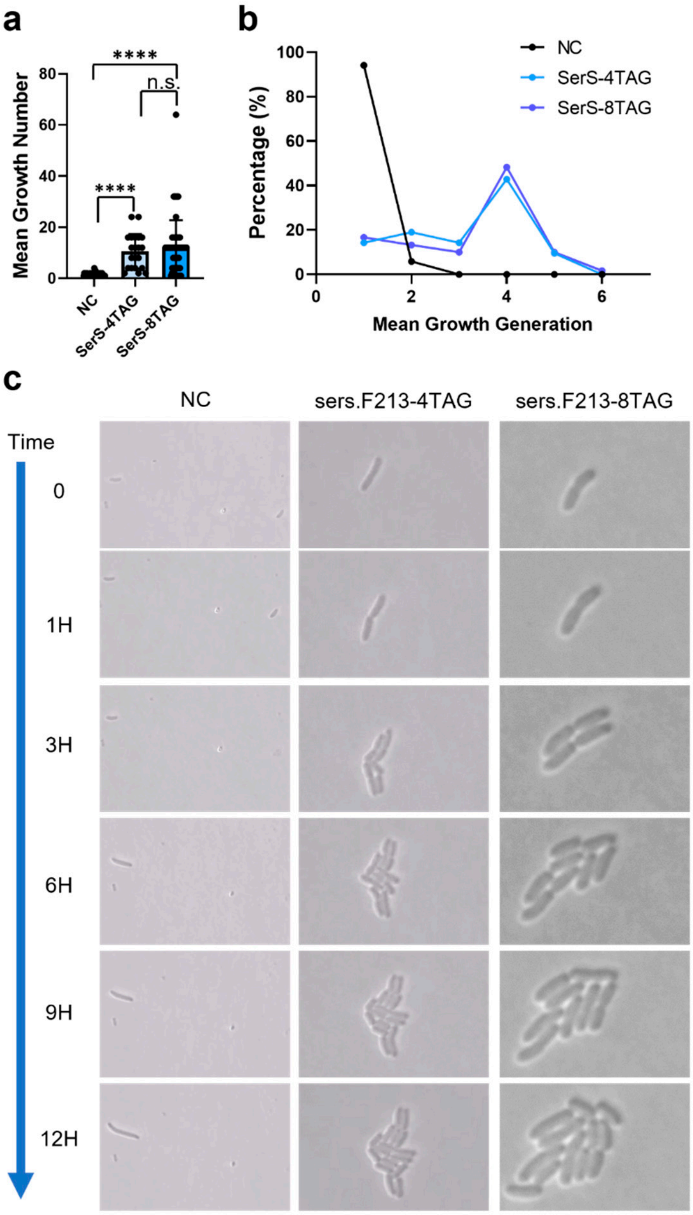

Figure S6. Functional validation of TAG codon optimization in SerS.F213 FRO rescue modules.

Comparative finite replication analysis of SerS.F213 FROs engineered with 4-TAG versus 8-TAG codon insertions in rescue modules, quantified by mean growth number (a) and distribution of generations (b). The SerS.F213-engineered FROs with 4-TAG and 8-TAG both demonstrated similar finite reproductive capacity, sustaining four generations. (mean ± SD; ****P < 0.0001, two-tailed Student's t-test). (mean ± SD; ****$P < 0.0001$, two-tailed Student's t-test). (c) Microscopic visualization of finite replication in optimized SerS.F213 FROs. Microscopic observation is imaged by the optical microscope (Nikon Inverted Research Microscope ECLIPSE Ti2-E/Ti2-E/B). The bacterial growth was observed and filmed every 0, 1, 3, 6, 9, and 12 h.

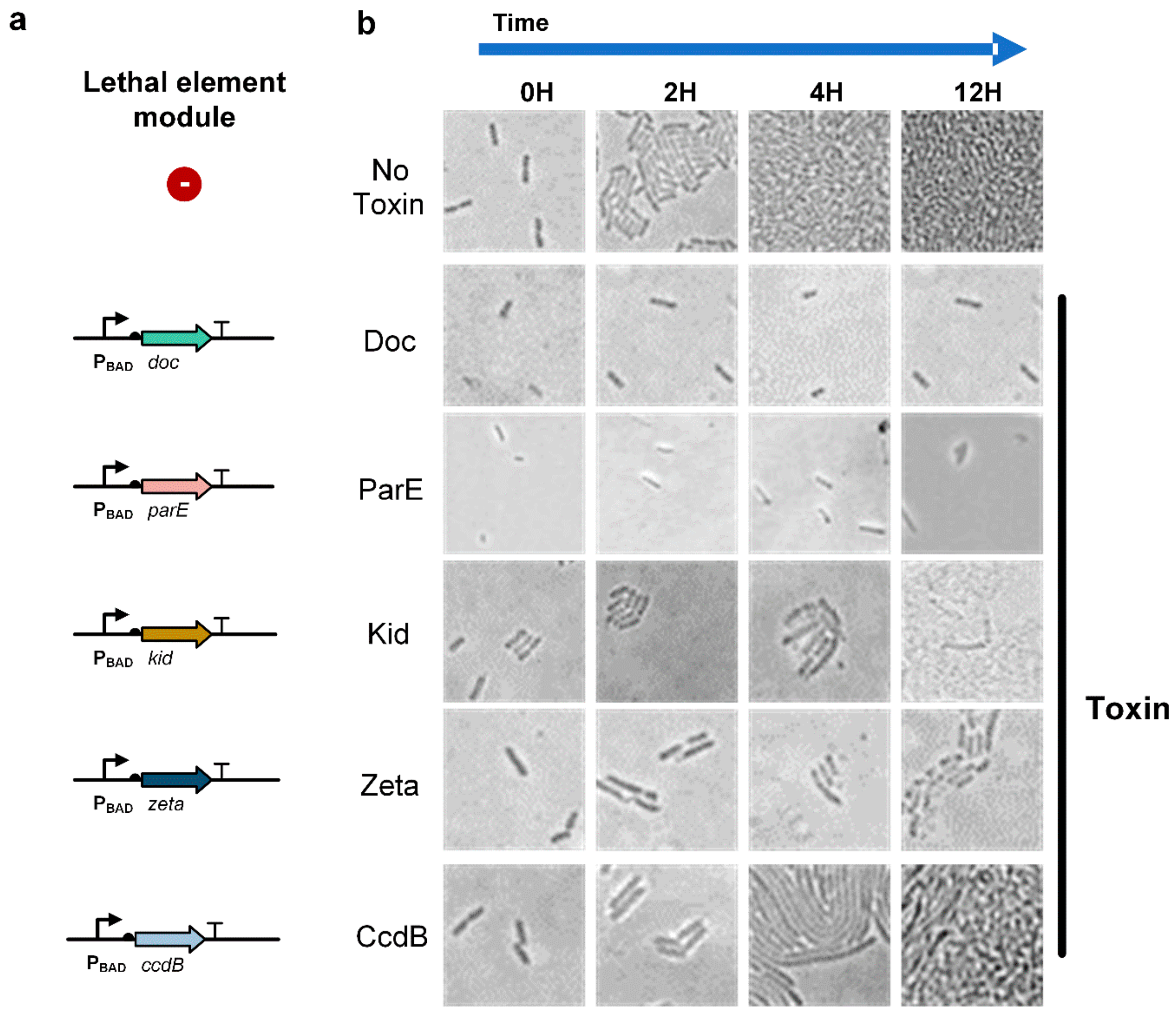

Figure S7. Screening of toxin-mediated suicide modules.

(a) Design of toxin-inducible systems. Schematic representation of genetic circuits containing toxin genes regulated by inducible promoters. (b) Growth phenotype analysis. Microscopic observation of bacterial strains cultured under induced toxin expression conditions. Microscopic observations are imaged by the optical microscope (Nikon Inverted Research Microscope ECLIPSE Ti2-E/Ti2-E/B). The bacterial growth was observed and filmed every 0, 2, 4, and 12 h.

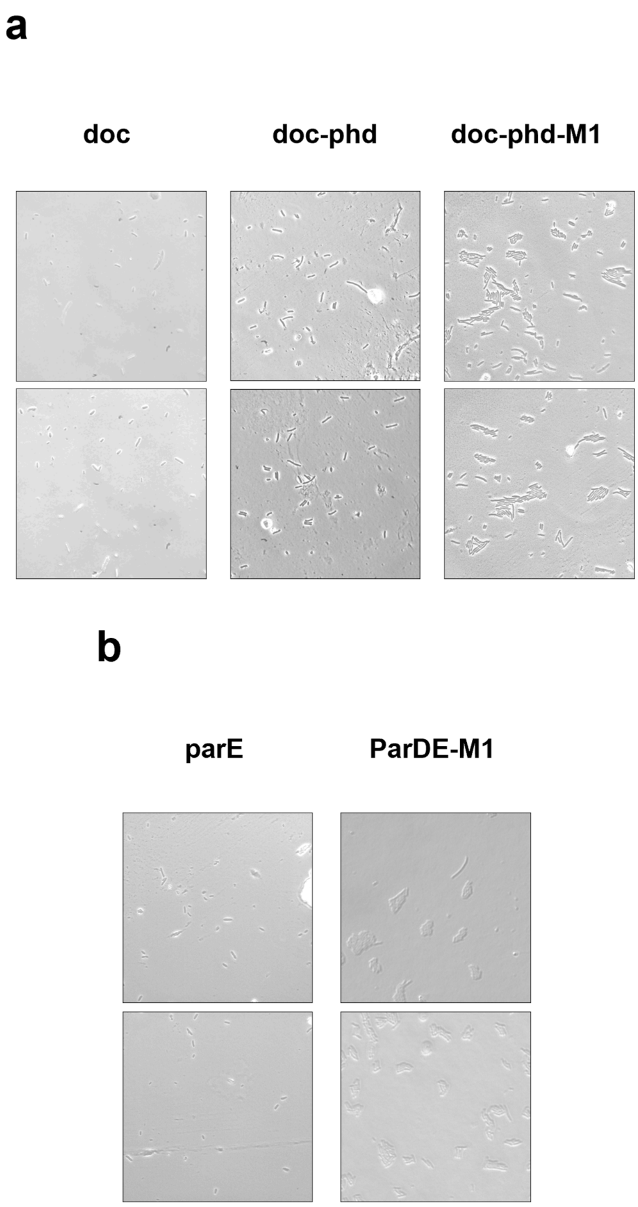

Figure S8. Representative microscopy of finite replication of FROs based on TA systems through TAG insertions in antitoxin *phd* (a) and *parD* (b).

Microscopic observations are imaged by the optical microscope (Nikon Inverted Research Microscope ECLIPSE Ti2-E/Ti2-E/B). The bacterial growth was observed and filmed at 12 hours.

Supplementary Video S1: Time-lapse observation of the typical single colony in the SerS.F213 FRO.

Continuous observations are imaged by the optical microscope (Nikon Inverted Research Microscope ECLIPSE Ti2-E/Ti2-E/B) corresponding to Fig. 2d. SerS.F213 FROs were engineered through the insertion of a rescue module that expresses the storage protein RFP, which carries eight TAGs. The video is played at a speed of 25 frames per second.

Supplementary Video S2: Time-lapse observation of a single colony in the SerS.F213-NC sample.

Continuous observations are imaged by the optical microscope (Nikon Inverted Research Microscope ECLIPSE Ti2-E/Ti2-E/B) corresponding to Fig. 2d. The SerS.F213-NC sample was constructed by pairing the ncAA module with the suicide module, in which dual TAG stop codons were site-specifically introduced into the essential gene *serS*. The video is played at a speed of 25 frames per second.

Supplementary Video S3: Time-lapse observation of the typical single colony in the blank control cells.

Continuous observations are imaged by the optical microscope (Nikon Inverted Research Microscope ECLIPSE Ti2-E/Ti2-E/B) corresponding to Fig. 2d. The video is played at a speed of 25 frames per second.

References

Abe, Y., Jo, T., Matsuda, Y., Matsunaga, C., Katayama, T., Ueda, T., 2007. Structure and Function of DnaA N-terminal Domains. Journal of Biological Chemistry. 282, 17816-17827.

Galindo-Feria, A. S., Notarnicola, A., Lundberg, I. E., Horuluoglu, B., 2022. Aminoacyl-tRNA Synthetases: On Anti-Synthetase Syndrome and Beyond. Frontiers in Immunology. 13.

Ha, S., Gross, B., Walker, S., 2001. E. Coli MurG: a paradigm for a superfamily of glycosyltransferases. Current drug targets. Infectious disorders. 1, 201-13.

Jiang, Y., Chen, B., Duan, C., Sun, B., Yang, J., Yang, S., Kelly, R. M., 2015. Multigene Editing in the Escherichia coli Genome via the CRISPR-Cas9 System. Applied and Environmental Microbiology. 81, 2506-2514.

Lajoie MJ, R. A., Goodman DB, Aerni HR, Haimovich AD, Kuznetsov G, Mercer JA, Wang HH, Carr PA, Mosberg JA, Rohland N, Schultz PG, Jacobson JM, Rinehart J, Church GM, Isaacs FJ., 2013. Genomically recoded organisms expand biological functions. . Science. Oct 18;342(6156):357-60.

Liu, X., Jiang, L., Li, J., Wang, L., Yu, Y., Zhou, Q., Lv, X., Gong, W., Lu, Y., Wang, J., 2014. Significant Expansion of Fluorescent Protein Sensing Ability through the Genetic Incorporation of Superior Photo-Induced Electron-Transfer Quenchers. Journal of the American Chemical Society. 136, 13094-13097.

Mandell, D. J., Lajoie, M. J., Mee, M. T., Takeuchi, R., Kuznetsov, G., Norville, J. E., Gregg, C. J., Stoddard, B. L., Church, G. M., 2015. Biocontainment of genetically modified organisms by synthetic protein design. Nature. 518, 55-60.

Salis, H. M., 2011. The Ribosome Binding Site Calculator. Synthetic Biology, Part B - Computer Aided Design and DNA Assembly. pp. 19-42.
